# The pathology of small airways disease in COPD: historical aspects and future directions

**DOI:** 10.1186/s12931-019-1017-y

**Published:** 2019-03-04

**Authors:** Andrew Higham, Anne Marie Quinn, José Eduardo D. Cançado, Dave Singh

**Affiliations:** 1 The University of Manchester Division of Infection, Immunity and Respiratory Medicine, School of Biological Sciences, Faculty of Biology, Medicine and Health, Manchester Academic Health Science Centre, Wythenshawe Hospital, Manchester University NHS Foundation Trust, Manchester, UK; 2 Department of Histopathology, Wythenshawe Hospital, Manchester University NHS Foundation Trust, Manchester, UK; 3 Santa Casa de Sao Paulo Medical School, Sao Paulo, Brazil; 4 Medicines Evaluation Unit, The Langley Building, Southmoor Road, Manchester, UK

**Keywords:** Bacteria, COPD, Emphysema, Exacerbations, Histopathology, Inflammation, Remodeling, Small airways disease

## Abstract

Small airways disease (SAD) is a cardinal feature of chronic obstructive pulmonary disease (COPD) first recognized in the nineteenth century. The diverse histopathological features associated with SAD underpin the heterogeneous nature of COPD. Our understanding of the key molecular mechanisms which drive the pathological changes are not complete. In this article we will provide a historical overview of key histopathological studies which have helped shape our understanding of SAD and discuss the hallmark features of airway remodelling, mucous plugging and inflammation. We focus on the relationship between SAD and emphysema, SAD in the early stages of COPD, and the mechanisms which cause SAD progression, including bacterial colonization and exacerbations. We discuss the need to specifically target SAD to attenuate the progression of COPD.

## Background

Chronic obstructive pulmonary disease (COPD) is caused by the harmful effects of cigarette smoking, or other noxious particles, resulting in persistent airflow limitation and symptoms including dyspnoea, cough and sputum production [1]. The burning of tobacco cigarettes produces a complex mixture of chemicals and gases capable of reaching the alveoli. The narrow diameter of the peripheral airways means that particles can more easily collide with the surface and cause damage compared to the larger airways [2]. The small airways have been defined as < 2 mm diameter and arise from the 4th – 13th generation of airway branching (taking trachea as 1st generation to alveoli as 23rd), but on average arise by the 8th [3]. It is estimated that 20% of small airways below 2 mm diameter comprise bronchi with elements of cartilage within their walls, while the remainder are bronchioles or alveolar ductal spaces. [4] Small airway disease (SAD) is a key feature of COPD, and has been studied extensively over many decades.

This article provides a comprehensive review of the pathology of SAD in COPD. We give a historical overview of key studies regarding the nature of SAD in COPD, and describe the various pathological features of SAD. We focus on clinically relevant topics, including the relationship between SAD and emphysema, and SAD in the early stages of COPD. We discuss the mechanisms which cause SAD progression, including bacterial colonization and exacerbations.

## A historical perspective: early observations of SAD in COPD

The relationship between airway disease and emphysema was first discussed in the nineteenth century. In 1819, René Laënnec recognised chronic bronchitis and emphysema as co-existing conditions [5], and William Gairdner (1850) discussed the impact of changes to distal airways and the relationship to emphysema [6], laying the foundations for our current understanding of the relationship between SAD and emphysema:

“*I am prepared, then, to maintain, that emphysema of the lung may, in all cases which I have witnessed, be satisfactorily accounted for by considering it as a secondary mechanical lesion, dependent on some condition of the respiratory apparatus leading to partially diminished bulk of the pulmonary tissue, and consequently disturbing the balance of air in inspiration.”*

Histopathological evidence of SAD in COPD gained momentum in the mid-twentieth century; Table 1 lists the major findings of studies spanning from 1953 to 1971 [7–14]. A striking feature of these studies is the heterogeneity of changes reported, including inflammation, fibrosis, narrowing, dilatation and obliteration of bronchioles. Leopold and Gough [8] reported narrowing of 60% of bronchioles supplying centrilobular emphysematous lesions. McLean [11] and later Hogg et al. [12] reported mucous plugging in small airways supplying emphysematous lesions. A loss of alveolar attachments, which radially connect to small airways like the spokes of a wheel, were also decreased in emphysematous tissue. This can reduce airway patency and render the airways more liable to collapse upon expiration. Overall, these changes can reduce airflow, increase gas trapping and thus reduce ventilatory capacity.

**Table 1:** The histopathological features of SAD in COPD specimens observed in studies spanning from 1953 to 1971

Author(s)	Histopathological features
Spain and Kaufman (1953) [7]	Inflammation, fibrosis and narrowing
Leopold and Gough (1957) [8]	Inflammation, narrowing, dilatation and obliteration
McLean (1958) [11]	Dilatation, inflammation and mucous plugging
Anderson and Foraker (1962) [9]	Narrowing and reduced alveolar attachments
Pratt et al. (1965) [10]	Reduced alveolar attachments
Hogg et al. (1968) [12]	Inflammation, mucous plugging and obliteration
Bignon et al. (1969) [16]	Inflammation and narrowing
Matsuba and Thurlbeck (1971) [14]	Narrowing and obliteration

In 1965, Macklem et al. measured bronchial pressures during respiratory maneuvers in patients with emphysema and healthy controls [15]. The small airways were identified as a location causing airflow obstruction in emphysema patients. These observations were expanded in the landmark study by Hogg, Macklem and Thurlbeck in 1968 using a retrograde catheter technique in excised lungs from five controls and seven emphysema patients [12]. The small airways (< 2 mm diameter) accounted for approximately 25% of the total airway resistance in healthy controls. The total airway resistance was increased in lungs from emphysema patients compared to controls, mainly due to increased small airways resistance, increasing by up to 40 fold. This can be explained by resistance changes having an inverse relationship with the fourth power of the decrease in airway radius [16]. Mucous plugging, inflammation, fibrosis and obliteration of small bronchioles were histopathological features in these patients. The authors introduced the term “small airways disease” to describe these changes.

The high level of heterogeneity in tissue pathology was commented on by Heppleston and Leopold [17] who advocated the need to harmonise the timing of emphysema pathology studies by either examining specimens from well-established cases of disease, or initial lesions before more extensive lung destruction occurs. This insightful suggestion resonates with the current interest in studying “early COPD” [18]. Mead referred to the small airways as a “quiet” zone, where SAD leading to eventual airway obstruction confirmed by spirometry can go unnoticed for years [19], leading to delayed COPD diagnosis.

## Histopathological features of small airway disease in COPD

Detailed studies using a variety of histopathological and imaging techniques have increased our understanding of the nature of SAD in COPD. The key pathological abnormalities are now reviewed.

### Airway remodelling

**Fig. 1:**
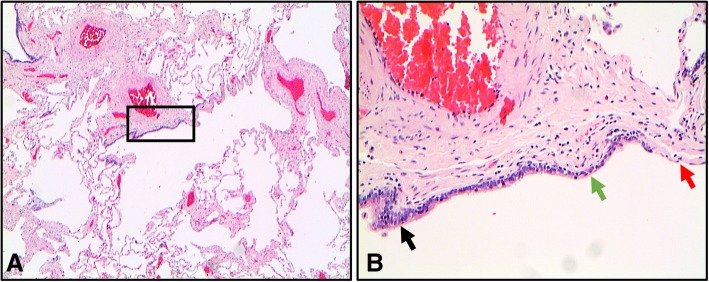
Transitioning epithelial structure in a respiratory bronchiole. **a** A bronchiole demonstrating transition from the columnar epithelium to cuboidal epithelium and finally to flattened alveolar epithelium (2X magnification). **b** An enlarged image of the inset from image A showing columnar epithelium (black arrow), cuboidal epithelium (green arrow) and flattened epithelium (red arrow) (10X magnification). Section stained with hematoxylin and eosin

The small airways can be regarded as airflow systems formed by membranous and respiratory bronchioles and alveolar ducts [20]. Membranous bronchioles include terminal bronchioles and are lined by columnar epithelial cells with cilia. The more distal respiratory bronchioles are lined by transitioning columnar to cuboidal epithelium and lead into alveolar ducts and alveolar spaces with flattened epithelium (Fig. 1) [21]. The bronchiolar wall can be divided into individual layers – the respiratory epithelium, basement membrane, lamina propria, smooth muscle (which is reduced in distal airways) and adventitia. Unlike the bronchial wall, seromucinous glands are not a usual feature and cartilage is not present within the bronchiolar structure.

**Fig. 2:**
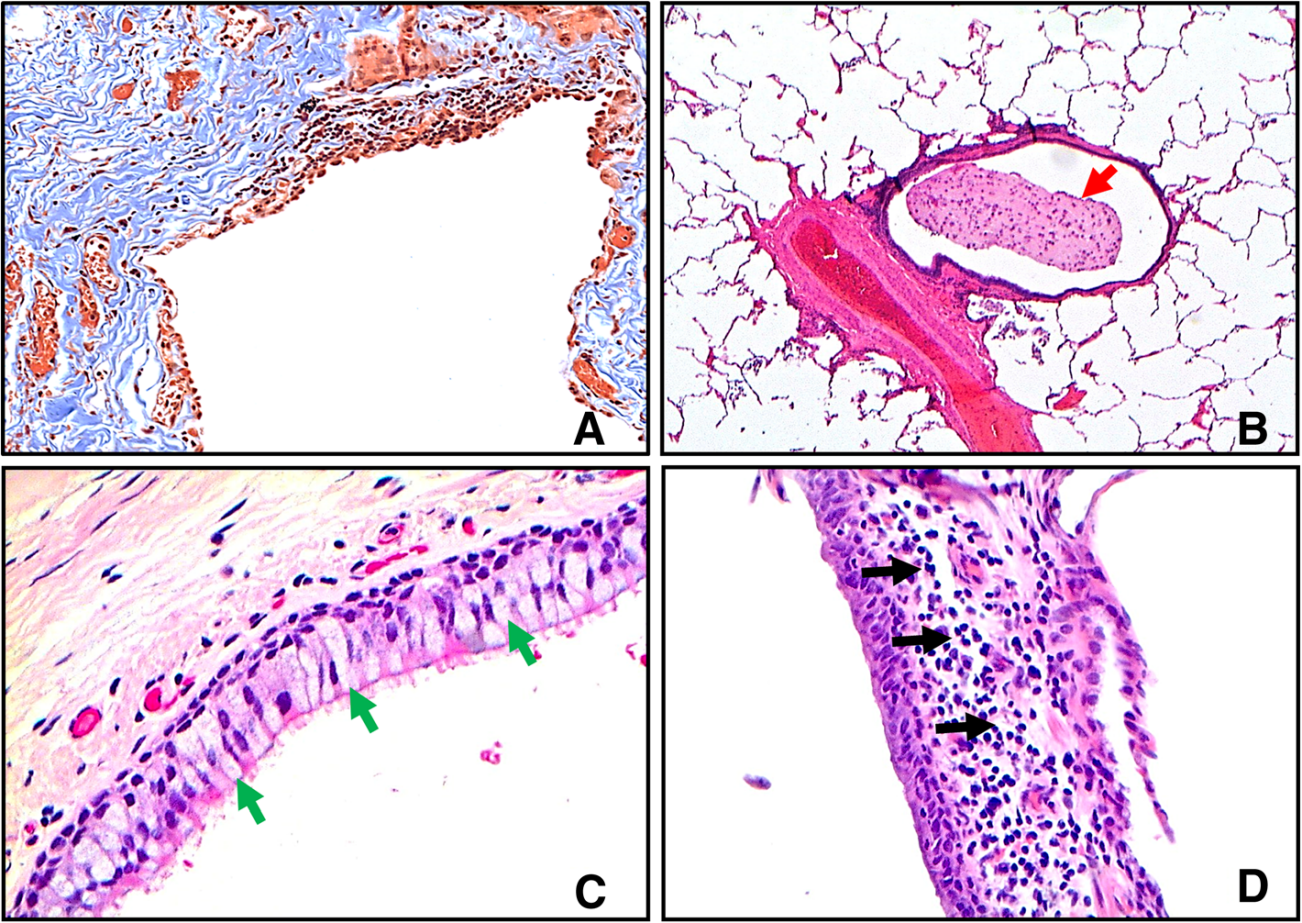
Histopathological features of small airways disease in COPD. **a** A COPD bronchiole with a thickened airway wall due to fibrotic remodeling and excessive deposition of collagen bundles (blue colouration). Section stained with Masson’s Trichrome (10X magnification). **b** A COPD bronchiolevascular bundle whereby the bronchiole contains a large intra-luminal mucous plug (red arrow) (2X magnification). Section stained with hematoxylin and eosin. **c** A COPD bronchiole with increased numbers of goblet cells (greenarrows) in the epithelial lining (20X magnification). Section stained with hematoxylin and eosin. **d** The wall of a COPD bronchiole with increased numbers of inflammatory cells (black arrows) (20X magnification). Section stained with hematoxylin and eosin

COPD small airways demonstrate marked remodelling, with the overall thickness of the airway wall increased compared to smokers without airflow limitation [22]. This increase in wall thickness stems from epithelial changes, mucoid plugs, increased density of inflammatory cells, smooth muscle hyperplasia and fibrosis (Fig. 2).

Airway remodelling is caused by wound healing in response to injury by cigarette smoke, viruses, bacteria etc. Wound healing is tightly regulated by the interaction between the immune response to these stimuli and the repair and remodelling of extracellular matrix by myofibroblasts [23]. Myofibroblasts are largely derived from sub-epithelial fibroblasts, surrounding epithelial cells which undergo epithelial mesenchymal transition (EMT) and circulating fibrocytes. The extracellular matrix (ECM) is then remodelled by proteolytic enzymes, such as metalloproteinases, to re-establish homeostatic balance of the tissue. However, due to the limited regenerative capacity of lung tissue, the process often leaves scarring. Myofibroblasts undergo apoptosis to prevent excessive scarring and fibrotic remodelling. Nevertheless, this process can lead to permanent alterations of tissue architecture [24].

The respiratory epithelium is composed broadly of four cell types; basal cells, ciliated cells, secretory cells and intermediate cells [25]. Following injury, re-epithelialisation with differentiated cell types is required to resume normal function. However, abnormal epithelial remodelling occurs in COPD with common changes including goblet cell metaplasia, basal cell hyperplasia and squamous metaplasia evident. These changes are more frequent in COPD compared to control small airways, [26, 27].

Basal cells act as the precursors for ciliated and secretory cells during re-epithelialisation [25, 28], but cigarette smoking alters the transcriptional program of basal cells, causing aberrant repair processes [28]. EMT appears to persist in COPD small airway epithelium; the myofibroblast markers, α-smooth muscle actin and vimentin, are increased in COPD small airway epithelial cells compared to non-smoking controls, and this negatively correlates with airflow limitation [29, 30]. The levels of the tight junction proteins, occludin, zona occludens-1 and E-cadherin are also reduced in COPD epithelium [31].

Changes to the bronchial epithelium can alter host-microbe interactions. For example, cigarette smoke upregulates the expression of platelet activating factor receptor promoting increased adherence of *nontypeable Haemophilus influenzae* (NTHi) and *Streptococcus pneumoniae* to bronchial epithelial cells [32]. Moreover, COPD bronchial epithelial cells produce lower amounts of the anti-microbial peptides, human defensin-2 and S100A7, when co-cultured with human rhinovirus, *Pseudomonas aeruginosa* or NTHi, compared to controls [33, 34]. There is also reduced mucosal immunoglobulin A (IgA) [26], that we will discuss later. The environmental conditions in COPD small airways appear to favour pathogenic microbial colonisation, which can drive further tissue inflammation and damage.

Fibrosis occurs in COPD small airways, with increased thickness of the individual mucosal compartments, and increased ECM protein deposition. Each mucosal layer is composed of a network of proteins including collagens, laminins and proteoglycans. Eurlings et al. showed significantly increased levels of total collagen in small airway walls of GOLD stage II and GOLD stage IV COPD patients compared to smoking controls [35]. Similarly, Kranenberg et al. demonstrated an increase in collagens I, III and IV in the basement membrane and collagens I and III in the lamina propria and adventitia of COPD patients compared to smoking controls [36]. Other proteins increased in COPD small airways mucosa include laminin, tenascin, and fibronectin [36, 37].

ECM proteins interact with immune cells regulating transmigration and cytokine secretion. For example, macrophages, neutrophils and lymphocytes all express laminin receptors [38]. Upon interaction with laminin 111 in vitro, neutrophils release higher amounts of tumour necrosis factor-alpha (TNF-α) and macrophage inflammatory protein-1 beta [39, 40] and macrophages release higher amounts of TNF-α and matrix metalloproteinase-9 [41, 42]. Interestingly, laminin has also been shown to positively regulate macrophage phagocytic function [42]. Altered ECM composition in COPD small airways may therefore influence immune cell behaviour.

ECM changes may also influence host-microbe interactions. For example, NTHi and *Moraxella catarrhalis* bind to laminin [43, 44] and *Moraxella catarrhalis* binds to collagen [45] . These structural proteins act as adhesion sites for bacteria, and have bactericidal activity. Specifically, collagen VI binds and kills *Morexella catarrhalis* [46]*.* How ECM changes in COPD impact host-microbe interactions requires further investigation. Increased adhesion combined with reduced bactericidal activity is inexorably linked with increased colonization, which are likely drivers of the early progress of SAD in COPD [18].

### Mucous plugging

The number of small airways with mucous plugging is increased in COPD patients, and corresponds with disease severity [22]. Furthermore, mucous occlusions of the small airways have been shown to be associated with early death in patients with severe emphysema treated by lung volume reduction surgery [47]. Mucous hypersecretion may cause small airway dysfunction by physically blocking airflow or by harbouring pathogenic microorganisms that promote further tissue inflammation and destruction. Cigarette smoking itself causes the pathological changes associated with mucous hypersecretion; there is greater mucous gland hypertrophy [48], goblet cell hypertrophy and hyperplasia in the large airways [49] and goblet cell hyperplasia and metaplasia in the small airways [22, 50, 51] in current smokers compared to non-smokers.

Mucous is a dynamic mixture of immune cells, cellular exudate, salts, lipids and proteins including enzymes and inflammatory mediators. The composition of mucous changes according to environmental cues. This is illustrated by the changes observed during COPD exacerbations whereby the numbers of sputum neutrophils and the levels of C-X-C motif chemokine ligand 8 (CXCL8), interleukin-17A (IL-17A), TNF-α, and IL-1β are increased compared to the stable state [52–54]. Mucins are glycoproteins found in mucous, which have viscoelastic properties that contribute to the biophysical character of mucous. Under normal conditions, the mucin content of sputum is around 2–5%. However, small increases in the levels of mucins can alter the biophysical properties of sputum [55], making it harder for ciliated epithelium to move the mucous along the respiratory tract.

Cigarette smoking shortens cilia length in the small airways and this is further worsened in COPD patients [56]. This may be due in part to an increase in ciliophagy, a process whereby cilia length is shortened in response to cigarette smoke exposure due to autophagy dependent mechanisms [57]. Cilia beat frequency is reduced in nasal cilia from COPD patients compared to healthy smokers [58]. Reduced cilia function will decrease the movement of mucous along the respiratory tract.

The evidence for a relationship between mucous hypersecretion and COPD itself (rather than active smoking) is less clear, with some studies showing either a difference or no difference in the number of goblet cells in the small airways between healthy smokers and COPD patients [26, 59–61]. Interrogating these studies in detail reveals that the selection of the study population, particularly the COPD group, is crucial to the outcome, and perhaps the presence of emphysema is important in this regard. Thurlbeck et al., reported that individuals with both chronic bronchitis *and* emphysema, but not individuals with chronic bronchitis only, had increased numbers of goblet cells compared to smoking controls [61]. This suggests that the extent of goblet cell metaplasia in the small airways may be related to the extent of parenchymal destruction. This requires further investigation.

Interestingly, the levels of the mucins mucin5AC (MUC5AC) and mucin5B (MUC5B) in sputum are elevated in COPD patients compared to healthy smokers and levels correlate with disease severity [62, 63]. Furthermore, epithelial expression of MUC5AC and intraluminal levels of MUC5B were increased in bronchioles devoid of sub-mucosal glands in COPD patients compared to smoking and non-smoking controls [60]. Known triggers of mucin production from airway epithelium relevant to COPD pathogenesis include rhinovirus [64–66], NTHi and *Pseudomonas aeruginosa* [67–69]. Mouse studies have demonstrated greater emphysema, inflammation and goblet cell metaplasia in the small airways following exposure to cigarette smoke and NTHi compared to either stimulus alone [70, 71]. It is likely that the combination of chronic cigarette smoking and microbial exposures contributes to SAD pathogenesis in COPD patients.

Increased goblet cell metaplasia is associated with a reduction in the levels of secretory IgA (SIgA) covering the surface of small airways in COPD patients [26]. Moreover, these airways had increased levels of bacterial 16S ribosomal RNA. SIgA is important for host defence at mucosal surfaces and so a loss will increase susceptibility to infection. IgA is produced by sub-epithelial plasma cells and transported across the bronchial epithelium to the apical surface by the polymeric Ig receptor (pIgR). Epithelial pIgR expression is reduced in COPD [72, 73] which may result in basolateral accumulation of IgA [74].

We have discussed evidence which highlights two important factors that may lead to the harbouring of pathogenic bacteria in COPD airways: reduced clearance of mucous and reduced anti-microbial activity of mucous. These changes may promote bacterial colonisation of the airways and increase inflammation. This has the potential to drive SAD progression, including at the earlier stages of COPD.

### Immune cell infiltration

Inflammation of the small airways due to smoking precedes fibrosis and tissue loss [75]. Some of the key advances in our knowledge of immune cell infiltration in COPD SAD came from publications in the late 1990’s and early 2000’s. These studies reported that the number of immune cells are further increased in the small airways of COPD patients compared to non-smokers and smokers without airflow limitation (Table 2). In two separate reports, Saetta et al. demonstrated an increase in the number of cluster of differentiation (CD) 68^+^ macrophages and CD8^+^ T-cells in the epithelium and CD8^+^ T-cells in the lamina propria of COPD small airways compared to non-smokers and smokers respectively [59, 76]. Furthermore, a later study from the same group showed that the numbers of intra-epithelial CD68^+^ macrophages and lamina propria CD4^+^ and CD8^+^ T-cells were higher in severe COPD patients compared to those with mild COPD [77]. In contrast, there was no increase in neutrophil numbers in COPD small airways in all three studies.

**Table 2:** Studies reporting immune cell infiltration in COPD SAD

Authors	Key findings						Area of quantification / Type of analysis	Patient groups
	Macrophages	Neutrophils	CD3^+^	CD4^+^	CD8^+^	CD20^+^		
Saetta et al. 1998 [76]	=	=	NQ	=	↑	NQ	Sub-epithelium excluding smooth muscle	COPD vs S
Saetta et al. 2000 [59]	↑	=	NQ	=	↑	NQ	Intra-epithelium	COPD vs NS
Turato et al. 2002 [77]	^a^↑	=	NQ	^b^↑	^b^↑	NQ	^a^Intra- and ^b^sub-epithelium	GOLD 3 vs S and GOLD 1
Hogg et al. 2004 [22]	^a^↑	^a^↑	NQ	^a^↑	^a, b^↑	^a, b^↑	^a^Number of airways +ve for cell type ^b^Accumulated volume	Increasing severity of disease
Baraldo et al. 2004 [82]	=	^a, b^↑	NQ	=	^a, b^↑	NQ	^a^Within smooth muscle ^b^percentage of airways +ve for cell type	COPD vs NS
Pilette et al. 2007 [80]	=	↑	↑	NQ	NQ	NQ	Intra-epithelium and lamina propria	COPD vs S
Battaglia et al. 2007 [83]	=	^a^↑	=	=	=	NQ	Lamina propria ^a^did not reach significance	COPD vs S
Kim et al. 2008 [84]	=	=	↑	=	=	NQ	Intra- and sub-epithelium	GOLD 4 vs GOLD 0
Olloquequi et al. 2010 [81]	NQ	NQ	=	=	↑	=	Intra- and sub-epithelium	GOLD2 vs NS
Isajevs et al. 2011[79]	↑	↑	NQ	NQ	↑	NQ	Intra- and sub-epithelium	COPD vs NS
Polosukhin et al. 2011 [27]	NQ	NQ	NQ	=	↑	NQ	Intra- and sub-epithelial	COPD vs NS
Polosukhin et al. 2017 [26]	↑	↑	NQ	NQ	NQ	NQ	Sub-epithelium	COPD vs NS GOLD 3/4 vs GOLD 1/2
Eapen et al. 2017 [78]	=	=	NQ	NQ	↑	NQ	Sub-epithelium	COPD vs NS

↑: increase in cell number; =: no difference

*NQ* not quantified, *NS* never smoker, *S* smoker without COPD, *GOLD* global initiative for chronic obstructive lung disease

"The meaning of a/b are described in the 'area of quantification/type of analysis' column."

In 2004, Hogg et al. took a different technical approach to the study of immune cell infiltration of the small airways [22]. The authors analysed small airways for macrophages, neutrophils, CD20^+^ B-cells, and CD4^+^ and CD8^+^ T-cells and separated their analysis into two endpoints: 1. extent of inflammation, represented by the number of airways positive for each immune cell; 2. degree of infiltrate, represented by the accumulated volume of each individual cell type within each airway. An increase in the total number of airways positive (extent of inflammation) was reported for macrophages, neutrophils, CD20^+^ B-cells, and CD4^+^ and CD8^+^ T-cells, which increased with COPD severity. In contrast, the accumulated volume of CD8^+^ T-cells and CD20^+^ B-cells only (degree of inflammation) was associated with COPD severity.

Following these seminal studies there have since been a number of publications reporting increased numbers of macrophages, neutrophils, CD4^+^ and CD8^+^ T-cells in COPD small airways [26, 27, 78–83]. These studies are shown in Table 2; it is clear there are some inconsistencies between studies with regard to which immune cells are increased in COPD small airways. There are two key technical factors to consider when interpreting these data. The first is the airway compartment where quantification is reported. For example, in two separate studies by Saetta et al., we can see that macrophages are increased in the epithelium [59] but not within the lamina propria [76] of COPD patients compared to controls. In contrast, neutrophils were not increased in the epithelium or lamina propria but were increased in the smooth muscle of COPD patients in a later study [82]. This highlights the need to consider individual airway compartments when assessing immune cell infiltration. The second key consideration is the number of inflammatory cells present varies greatly from airway-to-airway within the same patient, which may relate to extent and type of airway remodelling present, which again varies from airway-to-airway within the same patient [22]. There is evidence that certain types of pathology may be linked to specific immune cell infiltration. For example, the numbers of CD3^+^, CD4^+^ and CD8^+^ T-cells appear to correlate, albeit in some cases weakly, with goblet cell hyperplasia and squamous metaplasia in COPD small airways [27, 84]. An alternative approach is to combine the analysis of immune cell infiltration with assessment of remodelling to overcome this issue.

The numbers of lymphoid follicles associated with small airways increase with COPD severity [22]. However, some studies have failed to reproduce these findings, which may be related to sample size considerations or only mild to moderate COPD being investigated [85]. Another factor to consider is the use of corticosteroids, which is associated with lower numbers of lymphoid follicles [47]. Lymphoid follicles found in bronchiolar lung tissue are typically identified in the lamina propria, at the level of the smooth muscle, or deeper to this within the adventitia. Hence the name BALT, or bronchiolar-associated lymphoid tissue. These lymphoid follicles include reactive germinal centres with maturing B-lymphocytes, and are few in number in the healthy lung [86]. They aggregate in peribronchiolar tissue and within alveolar tissue under circumstances of inflammation.

### Relationship between SAD and emphysema

**Fig. 3:**
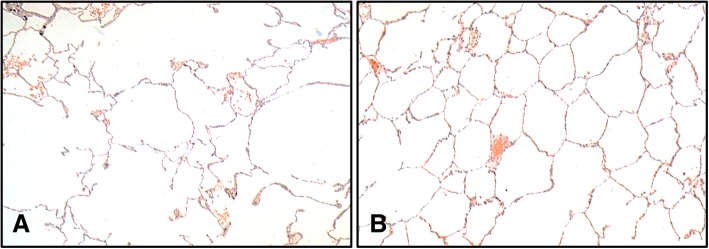
Smoking associated centrilobular emphysema. **a** An area of emphysematous lung with severe effacement of alveolar walls (4X magnification). **b** Normal lung with intact alveolar walls (4X magnification). Sections stained with hematoxylin and eosin

Centrilobular emphysema (Fig. 3) is strongly associated with chronic cigarette smoking and is distinct from panlobular emphysema (characteristically linked with α1-antitrypsin deficiency) and paraseptal emphysema [21]. Centrilobular emphysema affects the secondary pulmonary lobules, which are irregular, polyhedral units of lung structure defined by interlobular septa and supplied by a single pulmonary artery branch and pre-terminal bronchiole. Secondary pulmonary lobules contain 3–10 acini [20]. Each acinus includes the respiratory bronchioles, alveolar ducts and alveolar spaces.

Emphysematous destruction typically associated with smoking emanates from the centre of the lobule, hence the term centrilobular emphysema, first proposed by Leopold and Gough in 1957 [8]. Respiratory bronchioles stem from the centre of the pulmonary lobule, and these are the structures primarily affected by centrilobular (also termed centriacinar) emphysema. In contrast, panlobular emphysema affects all structures from distal alveoli to respiratory bronchioles, while paraseptal emphysema affects alveoli and alveolar ducts while sparing proximal structures.

How small airway remodelling, particularly fibrosis, relates to emphysema is somewhat paradoxical; on one hand there is thickening of tissue, whereas on the other there is tissue loss. A study by Gosselink et al. may go some way to help understand this process [87]. The authors used laser capture microdissection to examine the expression of 54 tissue-repair genes in small airways and the surrounding parenchyma. They found differential expression of these genes between the two areas with expression favouring parenchymal degradation surrounding the small airways. Why do these airways escape destruction? Perhaps these changes reflect the temporal nature of tissue remodelling in COPD. Gosselink et al suggest that some small airways undergo similar destruction to the parenchyma whereas others display a thickened profile.

Contemporary studies using micro-computed tomography have shown a reduction in the total number of terminal and first order respiratory (transitional) bronchioles in COPD patients compared to controls, with increased loss associated with disease severity [88, 89]; 90% of terminal bronchioles were obliterated in stage IV COPD lungs [88]. Koo et al. reported a reduction in the number of terminal and transitional bronchioles despite the absence of emphysema. In GOLD 1 patients, the reductions of terminal and respiratory bronchioles were 29 and 41% respectively and in GOLD 2 patients the reductions were 40 and 53% respectively. The remaining small airways had thickened walls and narrowed lumens due to mucoid plugs and collagenous deposits. The loss and remodelling of terminal and transitional bronchioles in lung tissue not affected by emphysema provides further evidence that SAD precedes emphysematous lesions. Perhaps collateral ventilation via intra-bronchiolar and intra-alveolar channels preserves distal gas exchange regions until eventually these also succumb.

The balance of proteinases and anti-proteinases is a classic concept in the pathogenesis of COPD [90]. Two recent murine studies have demonstrated the potential role of a disintegrin and a metalloproteinase domain-8 (ADAM8) and ADAM9 in cigarette smoke induced COPD-like pathology [91, 92]. In one study, cigarette smoke exposed ADAM8 knockout mice had greater lung inflammation, airspace enlargement and small airway goblet cell metaplasia compared to cigarette smoke exposed wildtype mice. Interestingly there was no difference in the extent of small airway fibrosis. ADAM8 expression was also reduced in COPD patients compared to controls [91]. In contrast, ADAM9 was shown to promote airspace enlargement, goblet cell metaplasia and fibrosis. Moreover, ADAM9 expression was higher in COPD patients compared to controls [92]. Considering the heterogeneous nature of COPD pathology, perhaps there are spatial and temporal differences in the balance of proteinases and anti-proteinases that determine the type of remodelling which occurs.

Exacerbations may play an important role with regard to remodelling processes. Exacerbations are an acute worsening of respiratory symptoms that require a change in treatment, such as oral steroids and / or antibiotics. These are often caused by viruses or bacteria [93]. Evidence is emerging showing rapid turnover of the ECM during exacerbations. For example, degradation fragments of collagens I, III, IV and VI, and the pro-form of collagen V are increased during COPD exacerbations [94, 95]. COPD exacerbations are associated with an increase in sputum neutrophils and neutrophil proteases [96]. There is increased expression and activity of matrix metalloproteinases in the bronchoalveolar lavage fluid of COPD patients during exacerbations [97], indicating dysregulation of proteinase activity in the distal lungs. Proteinases are involved in the turnover of the ECM and thus are inextricably linked to tissue destruction. They also have a role in mucin processing; mucin stability is highest at the start of a COPD exacerbation and this is associated with decreased neutrophil elastase activity and increased alpha 1 protease inhibitor activity [98]. COPD exacerbations may compound the effects of mucous plugging in SAD by reducing the mobility of mucous due to increased mucin concentration and viscosity.

## Future directions

**Fig. 4:**
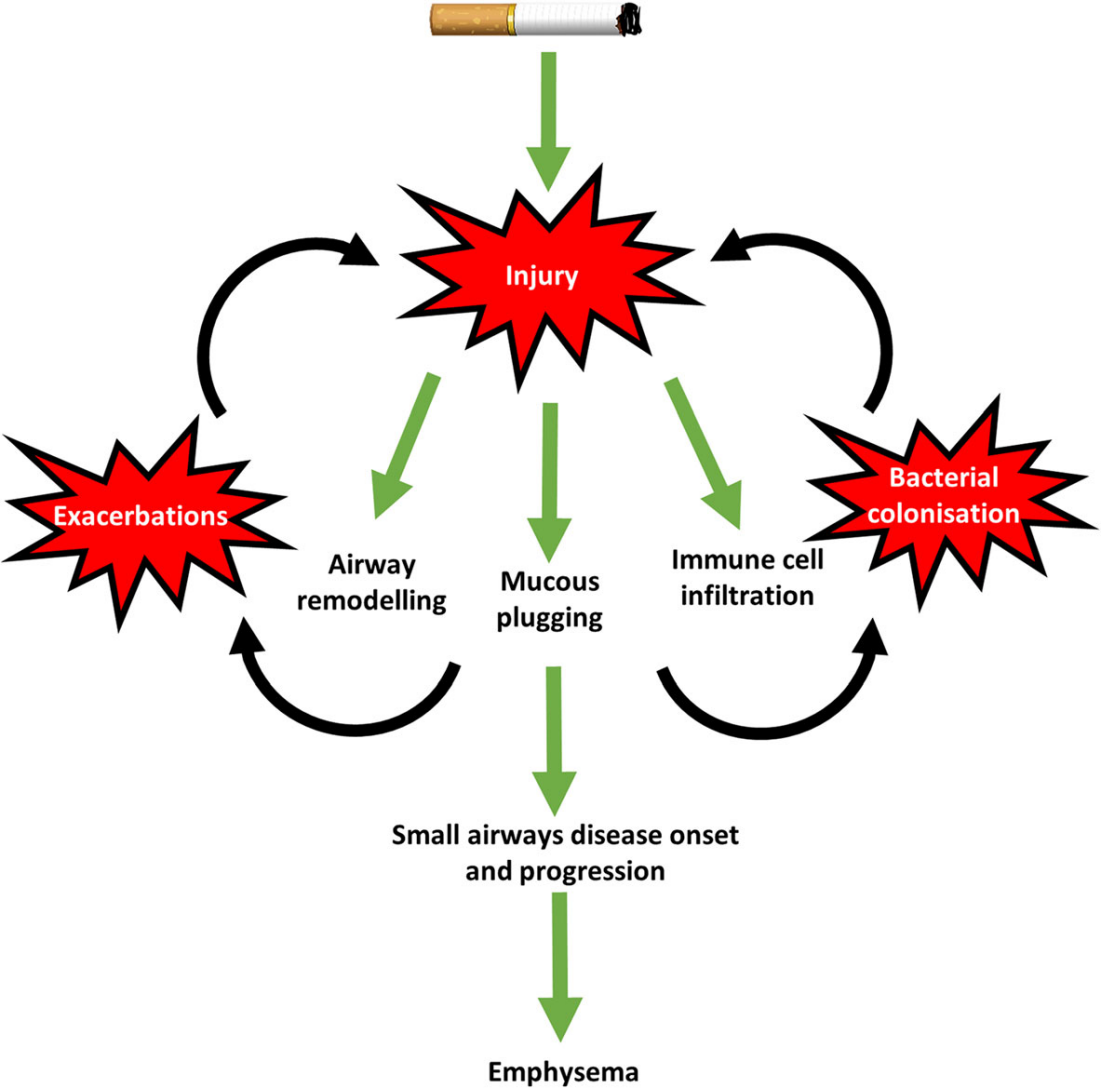
The mechanism of small airways disease onset and progression in COPD. Cigarette smoking causes injury to the small airways. In response, aberrant repair processes result in excessive airway remodelling, mucous plugging and immune cell infiltration. These contribute to the onset and progression of small airways disease, which precedes emphysema. These changes may lead to increased COPD exacerbations and bacterial colonization, which, in turn, may contribute to small airways disease progression and emphysema development

SAD is present at all stages of COPD, but we are becoming more aware of its importance in the earlier stages of COPD. SAD appears to be a precursor for the development of emphysema, and therapeutic strategies targeting the small airways in COPD may reduce the rate of emphysema progression. Such pharmacological targeting should be focused earlier rather than later in the natural history of COPD. We have discussed new insights into the progression of SAD including the role of bacteria in promoting SAD, and how exacerbations can promote inflammation and remodelling processes that are related to SAD (Fig. 4).

The aberrant repair process observed in COPD lungs may provide pathogenic bacteria with the opportunity to colonise the lungs, for example through reduced anti-microbial peptides and increased adhesion sites. Moreover, increased retention of mucous (due to increased expression of mucin proteins), combined with reduced anti-microbial properties of mucous (reduced SIgA), provide the bacteria with an ideal opportunity to colonise the lungs, already damaged by cigarette smoking induced disruption of the microenvironment. The impact of bacterial colonization may be chronic low grade inflammation over a period of years, or an acute, overwhelming level of inflammation which results in an exacerbation. Both will likely impact the emergence and progression of SAD.

This review highlights potential opportunities for pharmacological interventions that could disrupt the progression of small airway pathology in COPD. Many of these potential interventions target restoration of host anti-microbial defence. In contrast, we feel that the use of anti-inflammatory drugs that do not tackle persistent microbial infection is less likely to be effective, as these drugs will allow the persistence of pathogens that can stimulate the abnormal innate immune response that is a characteristic feature of COPD. Potential targets include restoration of anti-microbial defense by re-establishing SIgA function [26, 74], or modification of mucin characteristics in order to reduce viscosity and improve mucous clearance [62], which could also reduce bacterial colonization. Such strategies could involve altering the fate of epithelial basal cells to restore homeostatic function and avoid aberrant repair, including goblet cell metaplasia. The role of the Notch pathway, particularly during rhinovirus infection, may be important in determining goblet cell metaplasia [99]. Alternatively, modifying wound healing to reduce bacterial adhesion sites and improve bactericidal activity of the ECM holds potential. The role of ADAM8 and ADAM9 during the balance of protease-anti-protease biology have been discussed in this article and may prove useful targets to promote proper wound healing [91, 92].

## Conclusion

The origins of our understanding of the histopathology of SAD in COPD can be traced back to the early nineteenth century. Since then there has been a wealth of literature which has helped shape our knowledge of the key histopathological features of SAD. Future progress needs to focus on molecular mechanisms that drive the heterogeneity of COPD disease progression, as it is known that some patients can progress rapidly while others can remain relatively stable for years [100, 101]. While the role of smoking induced inflammation is well recognised, the contribution of damage to host defence mechanisms leading to bacterial colonization appears to be important. This interplay is likely to dictate small airway remodelling and destruction, and the development and progression of emphysema secondary to SAD.

## Abbreviations

ADAM8: A disintegrin and a metalloproteinase domain-8
ADAM9: A disintegrin and a metalloproteinase domain-9
BALT: Bronchiolar-associated tissue
CD: Cluster of differentiation
COPD: Chronic obstructive pulmonary disease
CXCL8: C-X-C motif chemokine ligand 8
ECM: Extracellular matrix
EMT: Epithelial mesenchymal transition
GOLD: Global initiative for chronic obstructive lung disease
IgA: Immunoglobulin A
IL-17A: Interleukin-17A
IL-1β: Interleukin-1beta
MUC5AC: Mucin 5 AC
MUC5B: Mucin 5B
NTHi: Nontypeable Haemophilus influenza
pIgR: Polymeric immunoglobulin receptor
SAD: Small airways disease
TNF-α: Tumour necrosis factor-alpha
